# Supplementary figures and images for: Identification, Analysis, and Confirmation of Seed Storability-Related Loci in Dongxiang Wild Rice (Oryza rufipogon Griff.)
Source: Genes (Basel). 2021 Nov 19;12(11):1831. doi: 10.3390/genes12111831 (PMC8622159; doi:10.3390/genes12111831)

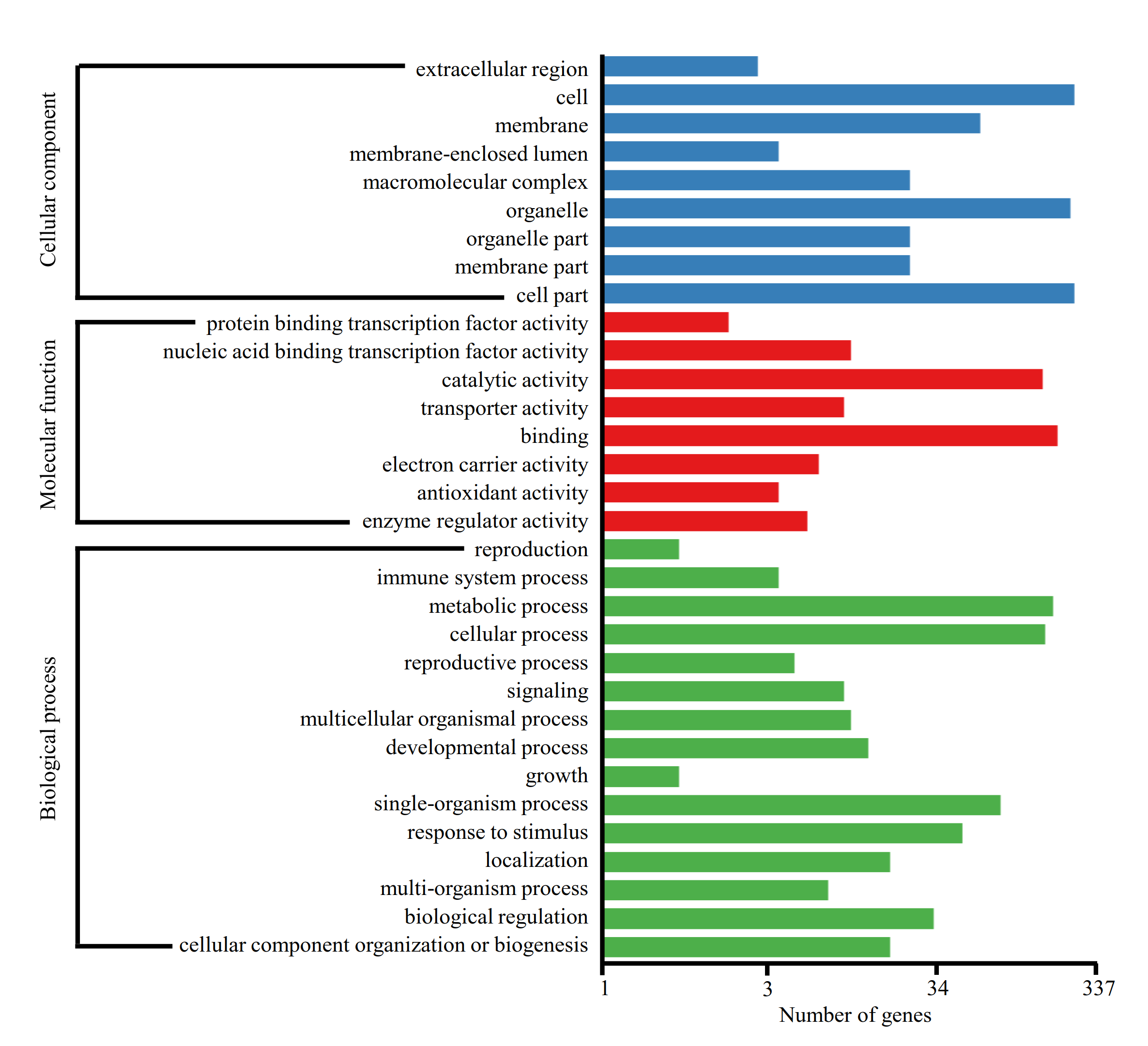

Supplement: Supplementary file 1 [file genes-12-01831-s001.zip › Supplementary/Figure S1.tif]

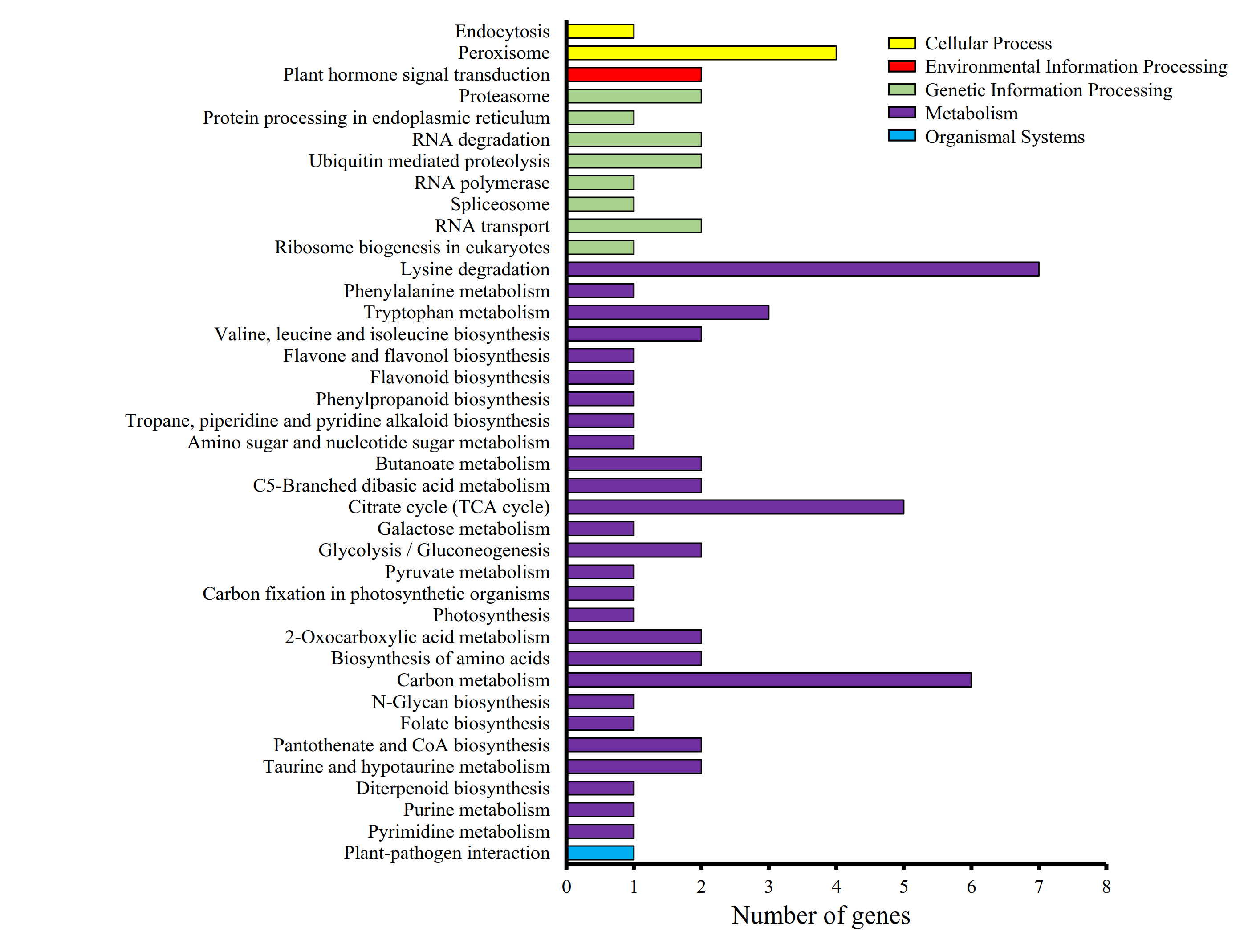

Supplement: Supplementary file 1 [file genes-12-01831-s001.zip › Supplementary/Figure S2.tif]
